# The importance of laboratory medicine in the medical student curriculum

**DOI:** 10.3402/meo.v20.30309

**Published:** 2015-12-17

**Authors:** Ishani Barai, Karishma Gadhvi, Pooja Nair, Sunila Prasad

**Affiliations:** 1 Faculty of Medicine, Imperial College London, London, UK; 2 Imperial College London, London, UK

The objective data provided by laboratory medicine underpins clinical practice in the areas of diagnosis, prognosis, and therapy. Molinaro et al. reported that approximately ‘60 to 70% of medical decisions are based on laboratory results’ (1). However, current literature states that medical education on laboratory testing is inadequate (2). In May 2008, the Centers for Disease Control and Prevention (CDC) highlighted that insufficient understanding of laboratory tests in the clinical setting leads to misinterpretation of results jeopardizing patient safety, and inefficient test selection augmenting costs per patient (3). Thus, why is more importance not placed upon teaching medical students about laboratory medicine?

Often, exposure to laboratory medicine is limited to lecture-based teaching in short pathology modules within the medical student curriculum. Predominantly, the teaching focuses on disease pathogenesis, with occasional references to laboratory data, rather than the intricacies of laboratory techniques. Some medical students may gain such exposure through intercalated BSc degrees, special study modules, and independent research activities. Therefore, a high degree of variability exists in laboratory experience amongst medical students. Moreover, a UK-based survey of medical graduates established that approximately 20% felt ‘less than competent’ in utilising laboratory testing (4). This emphasises the need to standardise laboratory medicine teaching across the medical school curricula.

The General Medical Council (GMC) states in its Tomorrow's Doctors guidelines that graduates should be able to ‘critically appraise the results of relevant diagnostic, prognostic and treatment trials and other qualitative and quantitative studies as reported in the medical and scientific literature’ (5). In the era of evidence-based medicine, an in-depth appreciation of laboratory techniques will enable medical students to better evaluate methodology in scientific literature. This can be further extended to daily medical practice, for instance being aware of strengths and limitations of various diagnostic approaches (6). This is particularly relevant to certain specialities such as haematology and infectious disease, which encompass both clinical and laboratory practices. The issue of laboratory training being of a lower priority has been brought to the forefront by haematology trainees, with the hope to instigate national recommendations and improve laboratory skills (7).

Our recent exposure to laboratory medicine in our respective intercalated BSc degrees has enabled us to value the role of laboratory techniques, in the context of our medical education so far. We believe that all medical students should have similar opportunities to receive formalised teaching in laboratory medicine. Possible approaches may involve e-learning modules, shadowing a laboratory scientist, dedicated group tutorials, short-term laboratory placements, or supervised projects. Challenges of formalising such teaching may include issues with timetabling, coordinating appropriate methods of assessment, and resource allocation. Encouragingly, Molinaro et al. and Smith et al. have outlined several successful approaches to laboratory medicine teaching in US medical schools, with minimal disruptions to the existing curricula (1, 2).

Whilst pilot studies and reviews are required to establish the most effective teaching method, prioritising laboratory medicine in medical education is likely to enormously benefit clinical decision-making and modern medical care.

*Ishani Barai* Faculty of Medicine Imperial College London London, UK Email: ishani.barai11@imperial.ac.uk*Karishma Gadhvi* Imperial College London London, UK*Pooja Nair* Imperial College London London, UK*Sunila Prasad* Imperial College London London, UK
